# Supplementary material for: The Characterization of the Tobacco-Derived Wild Tomato Mosaic Virus by Employing Its Infectious DNA Clone
Source: Biology (Basel). 2022 Oct 6;11(10):1467. doi: 10.3390/biology11101467 (PMC9598653; doi:10.3390/biology11101467)
Supplement: Supplementary file 1 [file biology-11-01467-s001.zip › biology-1906274-supplementary.pdf]

Supplementary information

The Characterization of the Tobacco-Derived Wild Tomato Mosaic Virus by Employing Its Infectious DNA Clone

Jinlong Yin[1], Xin Hong[1], Sha Luo[1], Jingquan Tan[1], Yuanming Zhang[1], Yanglin Qiu[1], Muhammad Faizan Latif[1], Tao Gao[2], Haijia Yu[2], Jingke Bai[3], Shujun Li[3], and Kai Xu[1*]

[1] Jiangsu Key Laboratory for Microbes and Functional Genomics, College of Life Sciences, Nanjing Normal University, Nanjing 210023, China

[2] Jiangsu Key Laboratory for Molecular and Medical Biotechnology, College of Life Sciences, Nanjing Normal University, Nanjing, 210023, China.

[3] Key Laboratory for Green Preservation & Control of Tobacco Diseases and Pests in Huanghuai Growing Area, Tobacco Research Institute, Henan Academy of Agricultural Sciences, Xuchang 461000, China

*To whom correspondence should be addressed at the Jiangsu Key Laboratory for Microbes and Functional Genomics, College of Life Sciences, Nanjing Normal University, Nanjing 210023, China; E-mail: xukai@njnu.edu.cn

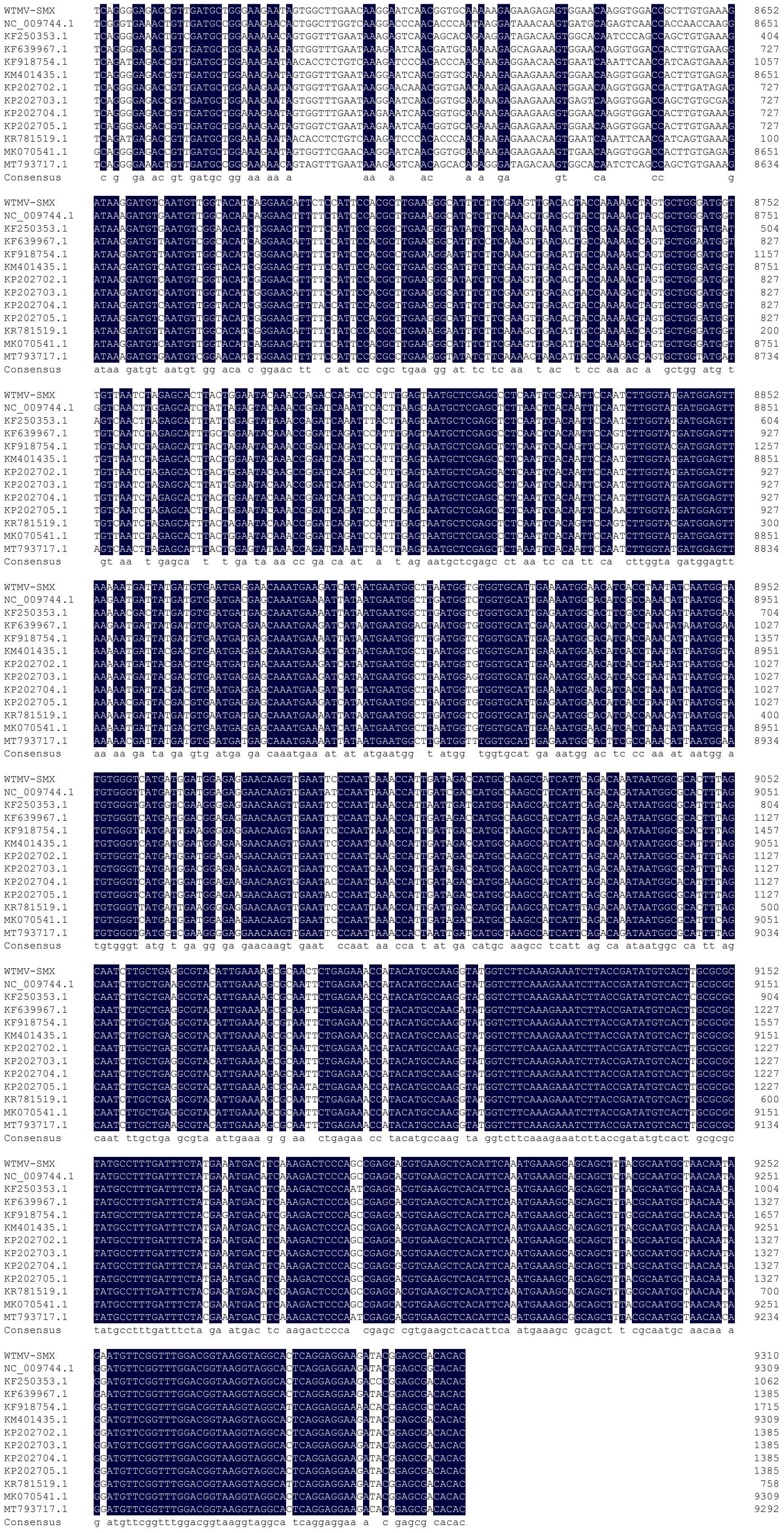

Supplementary Figure S1. Alignment of the partial genome of various WTMV isolates. Full or partial genome sequences of WTMV were aligned with Clustal W (gap-opening penalty = 10, gap-extension penalty = 5). The common region of the aligned WTMV sequences is shown.

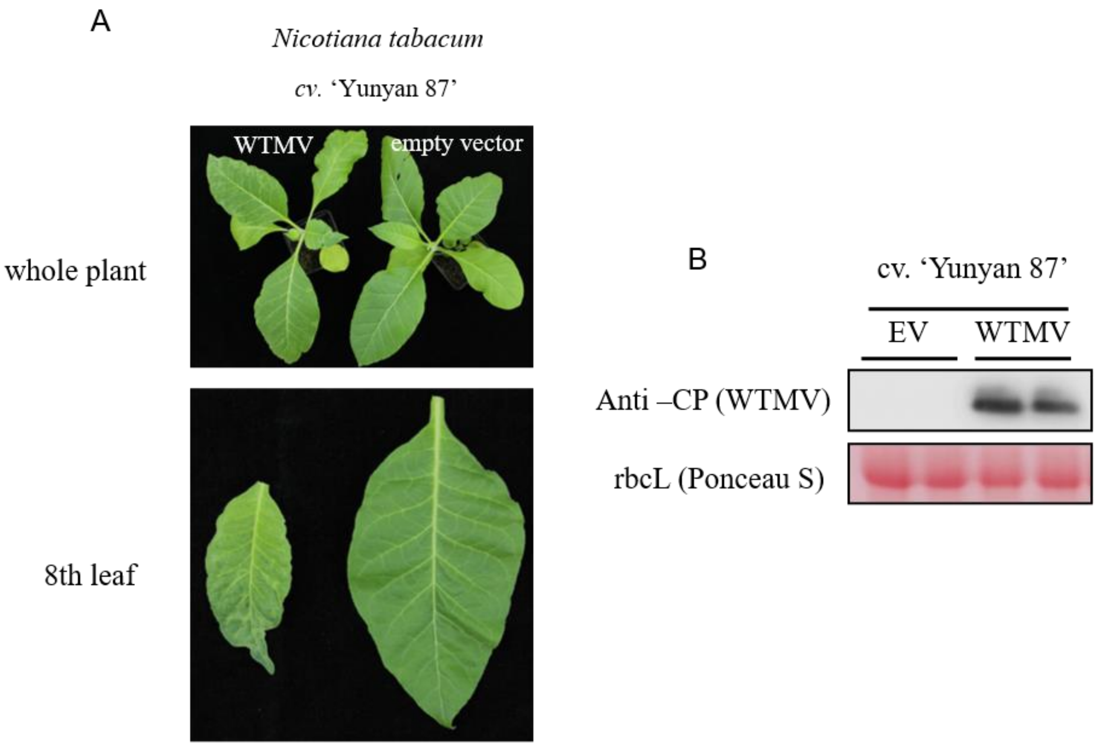

Supplementary Figure S2. WTMV infection on *N. tabacum* cv. 'Yunyan 87'. (A) *N. tabacum* cv. 'Yunyan 87' plants were agroinfiltrated with a WTMV infectious clone or the pCB301-304-CEN empty vector. The plant inoculated with WTMV showed severe systemic symptoms. Images were taken one-month post-agroinfiltration. Upper photograph: the whole plant of WTMV or empty-vector inoculated tobacco. Lower photograph: the eighth leaf of tobacco plants in corresponding treatment. (B) Western blotting analysis of upper leaves of tobacco plants in both treatments. Polyclonal antibodies of WTMV coat proteins were used to detect the presence of WTMV infection. Ponceau S stained rubisco large subunit (rbcL) is used as a loading control.

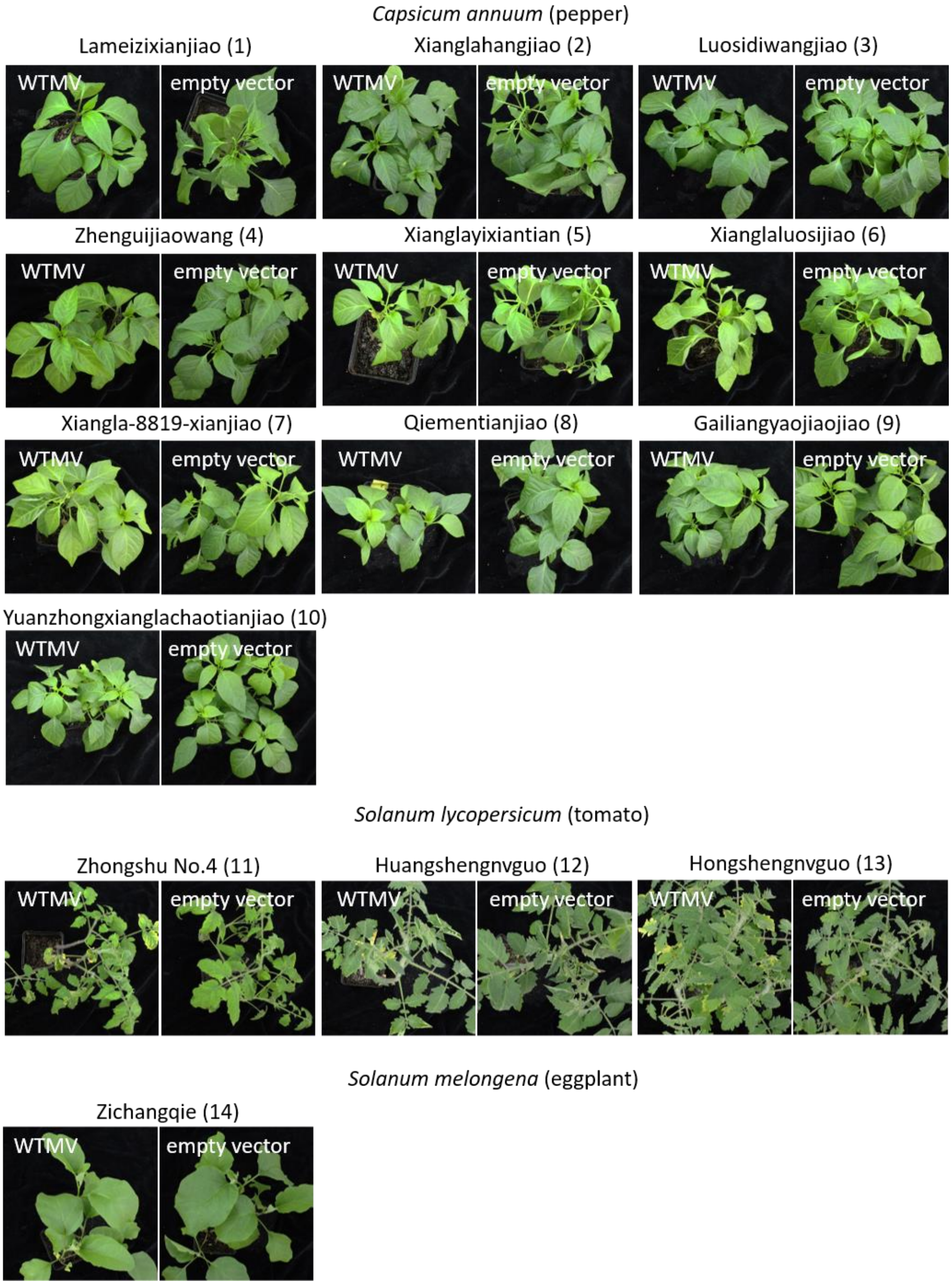

Supplementary Figure S3. Solanaceous plants inoculated with WTMV. Seedlings of the solanaceous plants agroinfiltrated with WTMV infectious clone or with pCB301-304-CEN empty vector. Images were taken 2 weeks after agroinfiltration. Variety names were listed above the corresponding photographs. The sources of the varieties are listed in Supplementary Table S1.

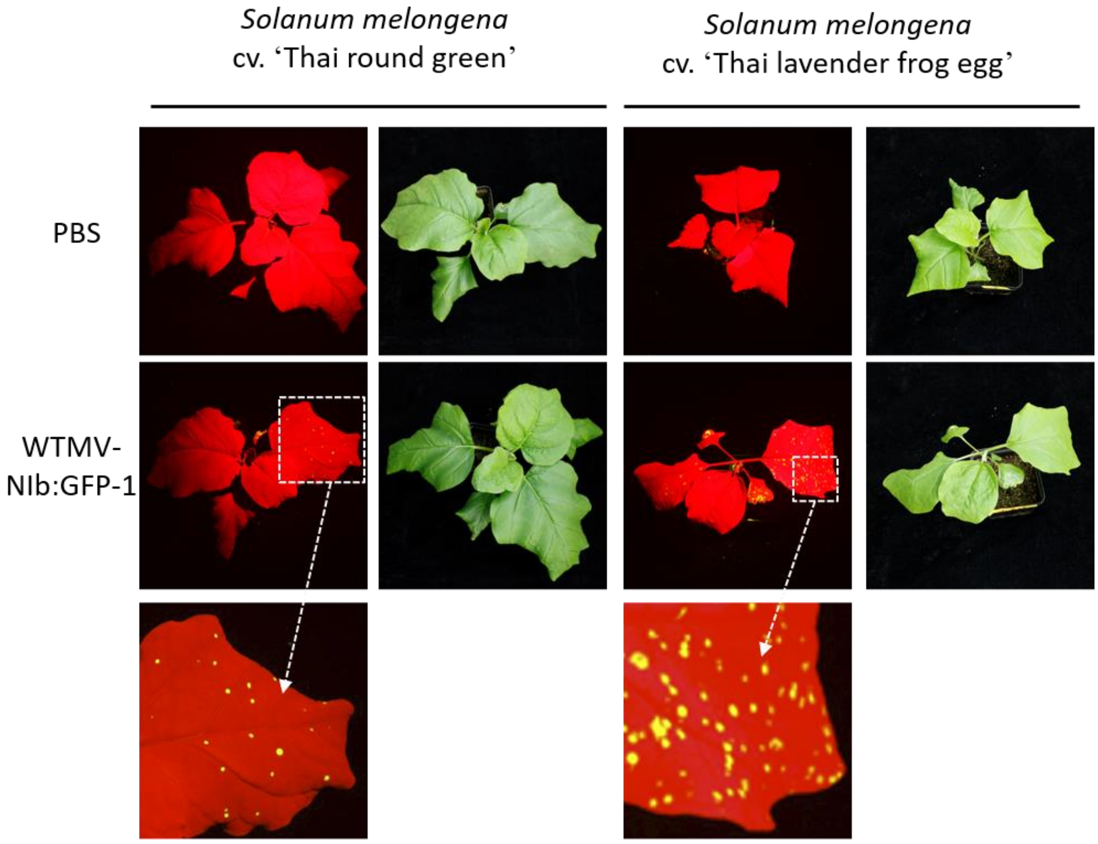

Supplementary Figure S4. Southeast Asian local *Solanum melongena* varieties inoculated with WTMV-NIb:GFP-1. Seedlings of *S. melongena* cv. 'Thai round green' and cv. 'Thai lavender frog egg' were inoculated with WTMV-NIb:GFP-1. WTMV infection was indicated by the EGFP fluorescence that appeared in systemic leaves two weeks after inoculation.

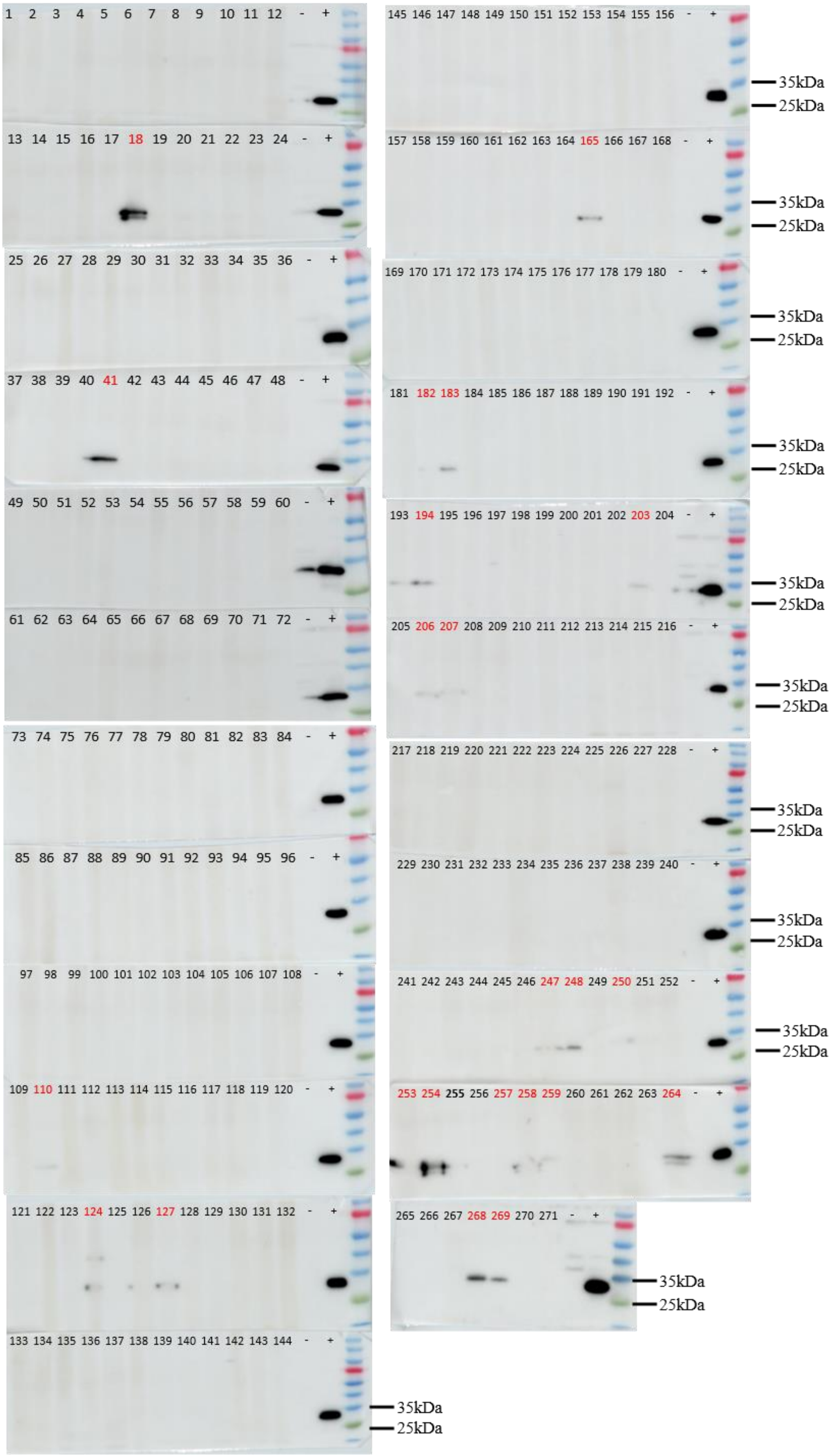

1 2 3 4 5 6 7 8 9 10 11 12 - +
13 14 15 16 17 18 19 20 21 22 23 24 - +
25 26 27 28 29 30 31 32 33 34 35 36 - +
37 38 39 40 41 42 43 44 45 46 47 48 - +
49 50 51 52 53 54 55 56 57 58 59 60 - +
61 62 63 64 65 66 67 68 69 70 71 72 - +
73 74 75 76 77 78 79 80 81 82 83 84 - +
85 86 87 88 89 90 91 92 93 94 95 96 - +
97 98 99 100 101 102 103 104 105 106 107 108 - +
109 110 111 112 113 114 115 116 117 118 119 120 - +
121 122 123 124 125 126 127 128 129 130 131 132 - +
133 134 135 136 137 138 139 140 141 142 143 144 - +
35kDa
25kDa
145 146 147 148 149 150 151 152 153 154 155 156 - +
35kDa
25kDa
157 158 159 160 161 162 163 164 165 166 167 168 - +
35kDa
25kDa
169 170 171 172 173 174 175 176 177 178 179 180 - +
35kDa
25kDa
181 182 183 184 185 186 187 188 189 190 191 192 - +
35kDa
25kDa
193 194 195 196 197 198 199 200 201 202 203 204 - +
35kDa
25kDa
205 206 207 208 209 210 211 212 213 214 215 216 - +
35kDa
25kDa
217 218 219 220 221 222 223 224 225 226 227 228 - +
35kDa
25kDa
229 230 231 232 233 234 235 236 237 238 239 240 - +
35kDa
25kDa
241 242 243 244 245 246 247 248 249 250 251 252 - +
35kDa
25kDa
253 254 255 256 257 258 259 260 261 262 263 264 - +
265 266 267 268 269 270 271 - +
35kDa
25kDa

Supplementary Figure S5. Western blotting analysis of the samples collected from fields in Henan province. A total of 271 samples were subjected to whole protein extraction and Western blotting using polyclonal antibodies of WTMV coat protein. Each sample was numbered. Positive and negative controls are protein samples from WTMV-infected or healthy tobacco leaves, respectively. Red-colored numbers are those selected for further RT-PCR analysis.

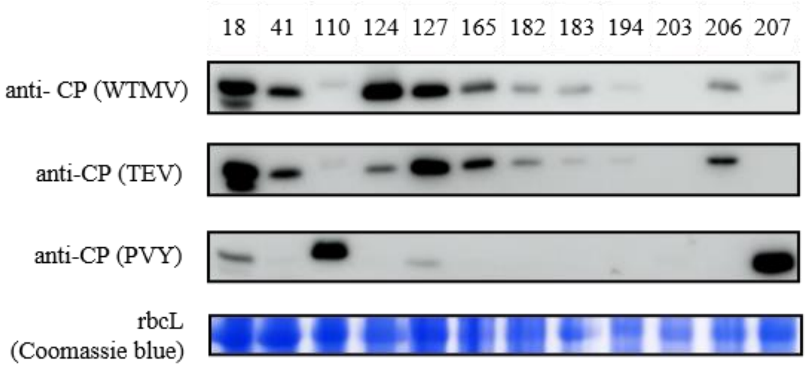

Supplementary Figure S6. Cross-reactivity of different polyclonal antibodies on tobacco samples. Previously identified WTMV-positive tobacco leaf samples were subjected to whole protein extraction and Western blotting against polyclonal antibodies of the coat proteins of WTMV, TEV, or PVY. Coomassie blue stained rbcL is shown to indicate protein loading.

Supplementary Table S1 Plant variety sources.

Species	Variety (Translated name)	Variety (Chinese name)	Source
Nicotiana benthamiana	-	-	lab source
Nicotiana tabacum	Samsun NN	-	lab source
Nicotiana tabacum	Zhongyan 100	中烟100	lab source
Nicotiana tabacum	Yunyan 87	云烟87	lab source
Solanum nigrum	-	-	寿光欣欣然园艺有限公司(Shouguang Xinran Horticulture Co., Ltd.)
Solanum torvum	-	-	C.H VIET NAM Co., Ltd.
Solanum melongena	Thai round green	-	lab source
Solanum melongena	Thai lavender frog egg	-	lab source
Capsicum annuum	Lameizixianjiao	辣妹子线椒	河北省泊头市永红种子有限公司(Botou Yonghong Seed Co., Ltd.)
Capsicum annuum	Xianglahangjiao	香辣杭椒	河北省泊头市永红种子有限公司(Botou Yonghong Seed Co., Ltd.)
Capsicum annuum	Luosidiwangjiao	螺丝帝王杂交一代	隆之喜种业（宿迁）有限公司(Longzhixi Seed Industry (Suqian) Co., Ltd.)
Capsicum annuum	Zhenguijiaowang	珍贵椒王	隆之喜种业（宿迁）有限公司(Longzhixi Seed Industry (Suqian) Co., Ltd.)
Capsicum annuum	Xianglayixiantian	香辣一线天	隆之喜种业（宿迁）有限公司(Longzhixi Seed Industry (Suqian) Co., Ltd.)
Capsicum annuum	Xianglaluosijiao	香辣螺丝椒	河北省泊头市永红种子有限公司(Botou Yonghong Seed Co., Ltd.)
Capsicum annuum	Xiangla-8819-xianjiao	香辣8819线椒	沧州禾硕农业科技有限公司(Cangzhou Heshuo Agricultural Technology Co., Ltd.)
Capsicum annuum	Qiementianjiao	茄门甜椒	沈阳市博农种业有限公司(Shenyang Bonong Seed Industry Co., Ltd.)
Capsicum annuum	Gailiangyaojiaojiao	改良羊角椒	沧州禾硕农业科技有限公司(Cangzhou Heshuo Agricultural Technology Co., Ltd.)
Capsicum annuum	Yuanzhongxianglachaotianjiao	原种香辣朝天椒	沧州禾硕农业科技有限公司(Cangzhou Heshuo Agricultural Technology Co., Ltd.)
Solanum lycopersicum	Zhongshusihao	中蔬四号	酒泉锐峰高科生态农业发展有限责任公司(Jiuquan Ruifeng High Tech Ecological Agriculture Development Co., Ltd.)
Solanum lycopersicum	Huangshengnvguo	黄圣女果	酒泉锐峰高科生态农业发展有限责任公司(Jiuquan Ruifeng High Tech Ecological Agriculture Development Co., Ltd.)
Solanum lycopersicum	Hongshengnvguo	红圣女果	酒泉锐峰高科生态农业发展有限责任公司(Jiuquan Ruifeng High Tech Ecological Agriculture Development Co., Ltd.)
Solanum melongena	Zichangqie	紫长茄	酒泉锐峰高科生态农业发展有限责任公司(Jiuquan Ruifeng High Tech Ecological Agriculture Development Co., Ltd.)

Supplementary Table S2 WTMV-positive tobacco samples collected from fields in Henan province.

Year	Number	Date	Location (location name in Chinese)
2020	#2	2020/6	Sanmenxia (三门峡)
2021	#41	2021/5/28	Xuchang, Xiangxian county, Wangluo town, shuanglouyan village (许昌, 襄县, 王洛, 双楼阎)
2021	#110	2021/6/22	Sanmenxia, Xiazhou district, Xiponao Village (三门峡, 陕州区, 西坡脑村)
2021	#124	2021/6/22	Luoyang, Yiyang county, Gaocun Village (洛阳, 宜阳, 高村)
2021	#127	2021/6/25	Xuchang, Xiangxian county, Wangluo town (许昌, 襄县, 王洛)
2021	#182	2021/6/30	Sanmenxia, lingbao city, wumu country (三门峡, 灵宝, 五亩乡)
2021	#183	2021/6/30	Sanmenxia, lingbao city, wumu country (三门峡, 灵宝, 五亩乡)

Supplementary Table S3 Primers used for virus detection

Target	Forward primer name	Forward primer sequence (5'-3')	Reverse primer name	Reverse primer sequence (5'-3')
WTMV	1275/WTMV/9131nt/F	CCGATATGTCACTTGCGCGC	1277/WTMV/3-end/R	AACGCCAACAAAGGAATAATGC
CMV	218/CMV-fny/CP/NdeI/F	GCCATATGGACAAATCTGAATCAACCAGTG	219/CMV-fny/CP/6xHis/EcoRI/R	CGGAATTCTTAATGATGATGATGATGGTGGACTGGGAGCACTCCAGACG
PVY	855/PYV-N/CP/6xHis/NcoI/F	ATGCCATGGGCCACCATCATCATCATCATAATGACACAATCGATGCAGGAGGAAG	856/PYV-N/CP/SalI/R	CGCGTCGACTCACATGTTCTTGACTCCAAGTAGAGTATGC
TEV	281/TEV/Frag3a/F	TTGGTTACATAGCAGAGGATGCTGGC	280/TEV/Frag2a/R	TGGAGATAGATAGTTTCCAGGTGATTGAAGTC
TMV	2652/TMV/check/F	GATCTAGCAAAGCGTCGTCT	2653/TMV/check/R	AATTTGCATCATCAGATATTCCAG
β-tubulin	2497/Tobacco/β-tubulin/qPCR/F	GGAGGTTACCGAGGCTGA	2498/Tobacco/β-tubulin/qPCR/R	GCATGTAGTCTTCCAAAG

Supplementary Table S4 Primers used for vector construction

Target vector	PCR product used for vector assembling	Intermediate PCR product	Forward primer name	Forward primer sequence (5'-3')	Reverse primer name	Reverse primer sequence (5'-3')	Template for PCR
pCB301-WTMV	WTMV-frag1	WTMV-frag1a	1717/WTMV/5-end/compatible/pCB301-linker/F	AGTTCATTTCATTTGGAGAGGAAAATAAAASAACTTCMMAACACATACAGAAAATC	1397/WTMV/797nt/R	GTTCCCTTCCAAGCGAATG	cDNA
		WTMV-frag1	44/pCB301/upstream/F	ACGTAAGGGATGACGCACAATCCCACTATCCTTCGCAAGACCCTTCCTCTATATAAGGAAGTTCATTTCATTTGGAGAGG	1397/WTMV/797nt/R	GTTCCCTTCCAAGCGAATG	WTMV-frag1a
	WTMV-frag2	WTMV-frag2	1396/WTMV/380nt/F	TAAATGATGAAAAGGAATCATTCTTGAG	1718/WTMV/Frag2a/R	CAGCATTGACCAATGAATCAAAGTG	cDNA
	WTMV-frag3	WTMV-frag3a	1719/WTMV/Frag3a/F	TCATGATGGACTTTGTTGC	1727/WTMV/3-end/polyA/pCB301-linker/R	GAGGTGGAGATGCCATGCCGACCCTTTTTTTTTTTTTTTTTTTTTTTTTTTTTTTTTTTTTTTTTTTTTTTTTTTAACGCCAACAAAGGAATAATGC	cDNA
		WTMV-frag3b	1719/WTMV/Frag3a/F	TCATGATGGACTTTGTTGC	11/SMV-frag5a/R-2	CTCTCCCTGACCCAGTGGCTCTCCCTTAGCCATCCGAGTGGACGTGCGTCCTCCTTCGGATGCCCAGGTCGGACCGCGAGGAGGTGGAGATGCCATGC	WTMV-frag3a
		WTMV-frag3	1719/WTMV/Frag3a/F	TCATGATGGACTTTGTTGC	12/SMV-frag5a/R-3	CCGGCAACAGGATTCAATCTTAAGAAACTTTATTGCCAAATGTTTGAACGATCGGGGAAATTCGAGCTCTCCCTGACCCAG	WTMV-frag3b
pCB301-WTMV-P1:GFP-1	WTMV-P1:GFP1-frag1	WTMV-P1:GFP1-frag1a	1066/pCB301/upstream-short/F	ACGTAAGGGATGACGCACAATCC	2338/WTMV/P1/R	TGAGAAATGAACCATACGCGTG	pCB301-WTMV
		WTMV-P1:GFP1-frag1b	2339/WTMVP1-GFP/F	GAGTTTACACGCGTATGGTTCATTTCTCAGTGAGCAAGGGCGAGGAGC	2341/GFP-FDVRHQS/R	GCGCTCTGATGTCTAACGTCGAACTTGTACAGCTCGTCCATGC	pGD-C-EGFP
		WTMV-P1:GFP1-frag1c	2340/FDVRHQS-WTMVHC/F	TTCGACGTTAGACATCAGAGCGCAGGTGAGAAGTTTTGGGAAG	1400/WTMV/1271nt/5RACE/R	AACTTAACGGCGTGTGGGAACGATGG	pCB301-WTMV
		WTMV-P1:GFP1-frag1	1066/pCB301/upstream-short/F	ACGTAAGGGATGACGCACAATCC	1400/WTMV/1271nt/5RACE/R	AACTTAACGGCGTGTGGGAACGATGG	WTMV-P1:GFP1-frag1a,WTMV-P1:GFP1-frag1b,WTMV-P1:GFP1-frag1c
	WTMV-P1:GFP-frag2	WTMV-P1:GFP1-frag2	2340/FDVRHQS-WTMVHC/F	TTCGACGTTAGACATCAGAGCGCAGGTGAGAAGTTTTGGGAAG	1718/WTMV/Frag2a/R	CAGCATTGACCAATGAATCAAAGTG	pCB301-WTMV
	WTMV-P1:GFP-frag3	WTMV-P1:GFP1-frag3	1719/WTMV/Frag3a/F	TCATGATGGACTTTGTTGC	1067/SMV-frag5a/R-3-short	CCGGCAACAGGATTCAATCTTAAGAAAC	pCB301-WTMV
pCB301-WTMV-P1:GFP-2	WTMV-P1:GFP2-frag1	WTMV-P1:GFP2-frag1a	1066/pCB301/upstream-short/F	ACGTAAGGGATGACGCACAATCC	2429/WTMV/GFP-HCpro/R	CCCAAAACTTCTCACCTGCAGATTGATGGTTAACAGCCTCCTTGTACAGCTCGTCCATGC	pCB301-WTMV-P1:GFP-1
		WTMV-P1:GFP2-frag1b	2428/WTMV/HCpro/F	GAGGCTGTTAACCATCAATCTGCAGGTGAGAAGTTTTGGG	1400/WTMV/1271nt/5RACE/R	AACTTAACGGCGTGTGGGAACGATGG	pCB301-WTMV
		WTMV-P1:GFP2-frag1	1066/pCB301/upstream-short/F	ACGTAAGGGATGACGCACAATCC	1400/WTMV/1271nt/5RACE/R	AACTTAACGGCGTGTGGGAACGATGG	WTMV-P1:GFP2-frag1a,WTMV-P1:GFP2-frag1b
	WTMV-P1:GFP-frag2	WTMV-P1:GFP2-frag2	2428/WTMV/HCpro/F	GAGGCTGTTAACCATCAATCTGCAGGTGAGAAGTTTTGGG	1718/WTMV/Frag2a/R	CAGCATTGACCAATGAATCAAAGTG	pCB301-WTMV
	WTMV-P1:GFP-frag3	WTMV-P1:GFP2-frag3	1719/WTMV/Frag3a/F	TCATGATGGACTTTGTTGC	1067/SMV-frag5a/R-3-short	CCGGCAACAGGATTCAATCTTAAGAAAC	pCB301-WTMV
pCB301-WTMV-NIb:GFP-1	WTMV-NIb:GFP-frag1	WTMV-NIb:GFP-frag1	1066/pCB301/upstream-short/F	ACGTAAGGGATGACGCACAATCC	1718/WTMV/Frag2a/R	CAGCATTGACCAATGAATCAAAGTG	pCB301-WTMV
	WTMV-NIb:GFP-frag2	WTMV-NIb:GFP-frag2	1719/WTMV/Frag3a/F	TCATGATGGACTTTGTTGC	2417/WTMV/NIb/R	TTGATGCCTTACATCAAAGTCATCATC	pCB301-WTMV
	WTMV-NIb:GFP1-frag3	WTMV-NIb:GFP1-frag3a	2418/WTMV/2707/F	TCGTGCTGAGCAACCAGAGC	2417/WTMV/NIb/R	TTGATGCCTTACATCAAAGTCATCATC	pCB301-WTMV
		WTMV-NIb:GFP1-frag3b	2419/WTMV/NIb-GFP/F	GTAGATGATGACTTTGATGTAAGGCATCAATCAGGGGTGAGCAAGGGCGAGGAGCTG	2420/WTMV/GFP-CP/R	CAACGGTCTCCCCGCTCTGATGTCTAACGTCGAAATCGTCCTTGTACAGCTCGTCCATGC	pGD-C-EGFP
		WTMV-NIb:GFP1-frag3c	2421/WTMV/GFP-CP/F	CGACGTTAGACATCAGAGCGGGGAGACCGTTGATGCTG	1067/SMV-frag5a/R-3-short	CCGGCAACAGGATTCAATCTTAAGAAAC	pCB301-WTMV
		WTMV-NIb:GFP1-frag3	2418/WTMV/2707/F	TCGTGCTGAGCAACCAGAGC	1067/SMV-frag5a/R-3-short	CCGGCAACAGGATTCAATCTTAAGAAAC	WTMV-NIb:GFP1-frag3a,WTMV-NIb:GFP1-frag3b,WTMV-NIb:GFP1-frag3c
pCB301-WTMV-NIb:GFP-2	WTMV-NIb:GFP-frag1	WTMV-NIb:GFP-frag1	1066/pCB301/upstream-short/F	ACGTAAGGGATGACGCACAATCC	1718/WTMV/Frag2a/R	CAGCATTGACCAATGAATCAAAGTG	pCB301-WTMV
	WTMV-NIb:GFP-frag2	WTMV-NIb:GFP-frag2	1719/WTMV/Frag3a/F	TCATGATGGACTTTGTTGC	2417/WTMV/NIb/R	TTGATGCCTTACATCAAAGTCATCATC	pCB301-WTMV
	WTMV-NIb:GFP2-frag3	WTMV-NIb:GFP2-frag3a	2418/WTMV/2707/F	TCGTGCTGAGCAACCAGAGC	2417/WTMV/NIb/R	TTGATGCCTTACATCAAAGTCATCATC	pCB301-WTMV
		WTMV-NIb:GFP2-frag3b	2426/WTMV/NIb-GFP/F	GTAGATGATGACTTTGATGTAAGGCATCAATCAGTGAGCAAGGGCGAGGAG	2427/WTMV/DVRHQS-CP/R	CGGTCTCCCCGCTCTGATGTCTAACGTCG	pGD-C-EGFP
		WTMV-NIb:GFP2-frag3c	2421/WTMV/GFP-CP/F	CGACGTTAGACATCAGAGCGGGGAGACCGTTGATGCTG	1067/SMV-frag5a/R-3-short	CCGGCAACAGGATTCAATCTTAAGAAAC	pCB301-WTMV
		WTMV-NIb:GFP2-frag3	2418/WTMV/2707/F	TCGTGCTGAGCAACCAGAGC	1067/SMV-frag5a/R-3-short	CCGGCAACAGGATTCAATCTTAAGAAAC	WTMV-NIb:GFP2-frag3a,WTMV-NIb:GFP2-frag3b,WTMV-NIb:GFP2-frag3c
pCB301-WTMV-RFP	WTMV-NIb:GFP-frag1	WTMV-NIb:GFP-frag1	1066/pCB301/upstream-short/F	ACGTAAGGGATGACGCACAATCC	1718/WTMV/Frag2a/R	CAGCATTGACCAATGAATCAAAGTG	pCB301-WTMV
	WTMV-NIb:GFP-frag2	WTMV-NIb:GFP-frag2	1719/WTMV/Frag3a/F	TCATGATGGACTTTGTTGC	2417/WTMV/NIb/R	TTGATGCCTTACATCAAAGTCATCATC	pCB301-WTMV
	WTMV-NIbRFP-frag3	WTMV-NIbRFP-frag3a	2418/WTMV/2707/F	TCGTGCTGAGCAACCAGAGC	2417/WTMV/NIb/R	TTGATGCCTTACATCAAAGTCATCATC	pCB301-WTMV-NIb:GFP-1
		WTMV-NIbRFP-frag3b	2648/RFP/WTMV/F	GTAGATGATGACTTTGATGTAAGGCATCAATCAGGGATGGTGTCTAAGGGCGAAG	2649/RFP/WTMV/R	ATTAAGTTTGTGCCCCAGTTT	pGD-C-tagRGFP
		WTMV-NIbRFP-frag3c	2650/RFP-CP/WTMV/F	CCCTAGCAAACTGGGGCACAAACTTAATGACGATTTCGACGTTAGACATC	1067/SMV-frag5a/R-3-short	CCGGCAACAGGATTCAATCTTAAGAAAC	pCB301-WTMV-NIb:GFP-1
		WTMV-NIbRFP-frag3	2418/WTMV/2707/F	TCGTGCTGAGCAACCAGAGC	1067/SMV-frag5a/R-3-short	CCGGCAACAGGATTCAATCTTAAGAAAC	WTMV-NIbRFP-frag3a,WTMV-NIbRFP-frag3b,WTMV-NIbRFP-frag3c
pCB301-TEV-GFP	TEV-GFP frag1	TEV-GFP frag1-1	277/TEV/Frag1a/pCB301-linker/F	aagttcatttcatttggagaggAAATAACAAATCTCAACACAACATATACAAAACAAACG	278/TEV/Frag1a/R	CGTCCCATGCATCCTTCAAAGCCTTTAC	TEV-GFP
		TEV-GFP frag1	44/pCB301/upstream/F	ACGTAAGGGATGACGCACAATCCCACTATCCTTCGCAAGACCCTTCCTCTATATAAGGAAGTTCATTTCATTTGGAGAGG	278/TEV/Frag1a/R	CGTCCCATGCATCCTTCAAAGCCTTTAC	TEV-GFP frag1-1
	TEV-GFP frag2	TEV-GFP frag2	279/TEV/Frag2a/F	CCATACATAATTGTCCTGGCAATAGTCTCCC	280/TEV/Frag2a/R	TTGGAGATAGATAGTTTCCAGGTGATTGAAGTC	TEV-GFP
	TEV-GFP frag3	TEV-GFP frag3	281/TEV/Frag3a/F	CTTGGTTACATAGCAGAGGATGCTGGC	282/TEV/Frag3a/R	CTGAAAATAAAGATTCTCAGTCGTTGGAATATCGTAATC	TEV-GFP
	TEV-GFP frag4	TEV-GFP frag4-1	283/TEV/Frag4a/F	GAGGGATGGCATGTATATACCAAAGCTAGAAG	284/TEV/frag4a/R1	GGAGGTGGAGATGCCATGCCGACCCTTTTTTTTTTTTTTTTTTTTTTTTTTTTTTTTTTTTTTTTTTTTTTTTTCTCGCACTACATAGGAGAATTAGACCG	TEV-GFP
		TEV-GFP frag4-2	283/TEV/Frag4a/F	TCATGATGGACTTTGTTGC	11/SMV-frag5a/R-2	CTCTCCCTGACCCAGTGGCTCTCCCTTAGCCATCCGAGTGGACGTGCGTCCTCCTTCGGATGCCCAGGTCGGACCGCGAGGAGGTGGAGATGCCATGC	TEV-GFP frag4-1
		TEV-GFP frag4	283/TEV/Frag4a/F	TCATGATGGACTTTGTTGC	12/SMV-frag5a/R-3	CCGGCAACAGGATTCAATCTTAAGAAACTTTATTGCCAAATGTTTGAACGATCGGGGAAATTCGAGCTCTCCCTGACCCAG	TEV-GFP frag4-2
